# Rapidly Developing Toxic Epidermal Necrolysis

**DOI:** 10.1155/2013/985951

**Published:** 2013-08-27

**Authors:** Viktoria Oline Barrios Poulsen, Jonas Nielsen, Troels Dirch Poulsen

**Affiliations:** ^1^Department of Anesthesia and Intensive Care Medicine, Copenhagen University Hospital, Roskilde Køgevej 7-13, 4000 Roskilde, Denmark; ^2^Intensive Care Unit 4131, Copenhagen University Hospital, Rigshospitalet, Blegdamsvej 9, 2100 Copenhagen, Denmark

## Abstract

Severe cutaneous reactions with potentially fatal outcomes can have many different causes. The Stevens-Johnson syndrome (SJS) and toxic epidermal necrolysis (TEN) are rare. They are characterized by a low incidence but high mortality, and drugs are most commonly implicated. Urgent active therapy is required. Prompt recognition and withdrawal of suspect drug and rapid intervention can result in favourable outcome. No further international guidelines for treatment exist, and much of the treatment relies on old or experimental concepts with no scientific evidence. We report on a 54-year-old man experiencing rapidly developing drug-induced severe TEN and presented multiorgan failure involving the respiratory and circulatory system, coagulopathy, and renal insufficiency. Detachment counted 30% of total body surface area (TBSA). SCORTEN = 5, indicating a mortality rate >90%. The patient was sedated and mechanically ventilated, supported with fluids and inotropes to maintain a stable circulation. Component therapy was guided by thromboelastography (TEG). The patient received plasmapheresis, and shock reversal treatment was initiated. He was transferred to a specialized intensive care burn unit within 24 hours from admittance. The initial care was continued, and hemodialysis was started. Pulmonary, circulatory, and renal sequelae resolved with intensive care, and re-epithelialization progressed slowly. The patient was discharged home on hospital day 19.

## 1. Introduction

The Stevens-Johnson syndrome (SJS) and toxic epidermal necrolysis (TEN) are rare but present severe skin manifestations. They are estimated to occur in 1–3 people/million/year in Europe and the USA [1, 2]. They are characterized by a low incidence but high mortality, and drugs are most commonly implicated in 80% of TEN cases [3, 4]. TEN is the most severe form of drug-induced skin reaction and is defined as epidermal detachment of >30% of total body surface area (TBSA). SJS presents with epidermal detachment of <10% of TBSA, whereas involvement of 10%–30% of TBSA is defined as SJS/TEN overlap [1, 2, 5]. Even in extensive disease, the hairy portion of the scalp is generally not affected [5]. The pathomechanism of drug-induced SJS/TEN is not completely understood but is assumed to be an immune-mediated skin reaction involving CD8 T cells, cytotoxic reactions, and delayed hypersensitivity [1, 6]. Histopathology is characterized by keratinocyte apoptosis followed by necrosis, which creates the basis for the pronounced epidermal erosion and detachment. There is a common agreement to consider this phenomenon as the manifestation of a dysregulated immune reaction against epithelial cells [2]. The systemic toxicity increases the risk for multiorgan failure. A genetic association in Han Chinese between the HLA-B∗1502, SJS, and carbamazepine has been confirmed, and other gene loci associations exist in the Asian populations. No genetic correlation has been shown in Europeans [2, 5, 7].

The cutaneous manifestation in TEN is preceded by a prodromal phase presenting fever and influenza-like symptoms, and within a couple of days, the generalized erythema formation of macules and finally epidermal detachment occur. The epithelial detachment may progress for 5 to 7 days after which a variable period of reepithelialization occurs. The wounds created by TEN are similar to second-degree burns [3] and lead to extreme pain, massive loss of fluid and protein, bleeding, evaporative heat loss with subsequent hypothermia, and infection.

A patient with TEN is suffering from “acute skin failure,” and the condition can be associated with major metabolic abnormalities, sepsis, multiorgan failure, pulmonary embolism, and gastrointestinal hemorrhage [5]. The mortality rate in TEN is 30–50%, but the outcome is difficult to predict [7]. A validated severity-of-illness score for TEN, abbreviated as SCORTEN (Table 1), was developed by Bastuji-Garin et al. [8] and may be used to estimate prognosis. SCORTEN has proven to be remarkably accurate in predicting mortality. Increasing age, significant comorbidities, and extensive cutaneous involvement are correlated with a poor prognosis [1, 2]. Debate still exists regarding the optimal treatment for SJS/TEN, and no randomised controlled trials or generally accepted guidelines exist. 

## 2. Case

A 54-year-old male was presented at the emergency room (ER) after an increasingly affected general condition during a couple of days. The ambulance call centre was previously contacted, and the patient was admitted to the hospital under the diagnosis of anaphylaxis. The patient was described as a well-functioning man, employed as innkeeper, and living with his wife and their newborn child. He suffered from hypertension and asthma and some years ago was found to be CMV-positive. He had no allergies. Three days prior to admittance, he had started treatment with penicillin prescribed by his general practitioner suspecting erysipelas located on the right lower leg. 

Upon arrival at the ER, the patient appeared septic presenting a generalized erythema. A large caliber peripheral intravenous line was placed, and intravenous epinephrine, intravenous antihistamine, and corticosteroid were given right away suspecting anaphylactic shock. Concurrently, fluid therapy was started. Paraclinical parameters revealed severe renal insufficiency and deranged coagulation.

The patient was acutely transferred to a more specialized hospital for the purpose of hemodialysis or plasmapheresis. Shortly before arrival, a rapid sequence induction and subsequent intubation were performed using alfentanil, propofol, and suxamethonium, and mechanical ventilation was started (PRVC, FiO_2_ 50%, PEEP 12). The patient was kept sedated with propofol combined with sufentanil and ventilated mechanically, and the hemodynamical instability was treated effectively with crystalloid fluids and continuous epinephrine infusion. Substituting component therapy was started guided by thromboelastography (TEG). Shock reversal therapy with methylprednisolone 120 mg × 2 was initiated.

A picture of septic shock was complicated by multiorgan failure involving lungs, circulation, kidneys, and coagulation. An arterial blood gas analysis at arrival at the intensive care unit exhibited severe metabolic acidosis with pH 7.21, base excess 12 mmol/L, HCO_3_^−^ 15 mmol/L, lactate 7.1 mmol/L, and plasma-glucose 5.1 mmol/L. An acute blood smear for schizocytes came out negative. Relevant cultures were ensured, and empiric antibiotic therapy was initiated including ciprofloxacin, meropenem, and metronidazole. Plasmapheresis was performed suspecting hemolytic uremic syndrome (HUS). Subsequently hemolysis was excluded, and drug reaction with eosinofilia and systemic symptoms (DRESS) was excluded due to a normal eosinophilic count.

**Figure 1:**
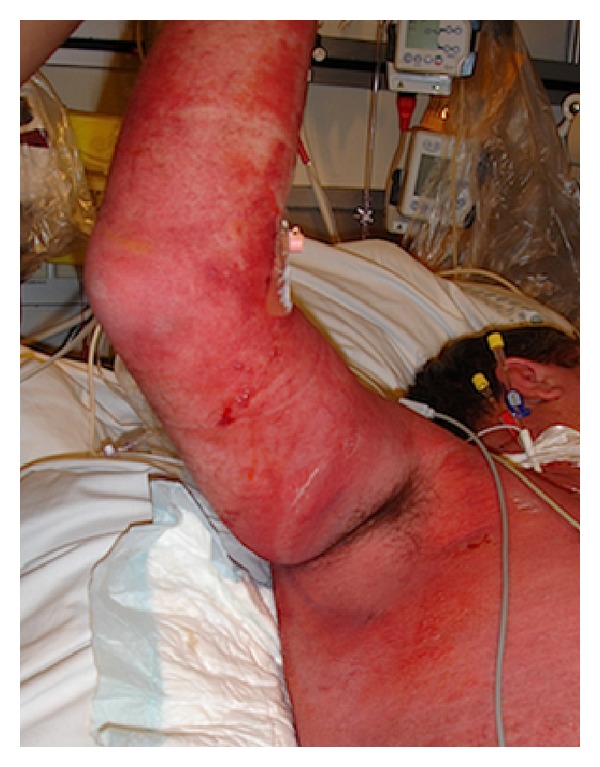
The patient presenting severe skin manifestations.

Within 12 hours, the patient developed fulminant purpura and erythema involving 80% TBSA with formation of macules and detachment of epidermis (positive Nikolsky) gravitational on extremities, dorsum and located to the genitofemoral areas (Figure 1). Petechiae were observed universally, as well as vulnerable mucosa orally. There was no skin affection corresponding to the face, hands, lower legs, and feet. The SCORTEN score was 5 equivalent to >90% mortality rate (Table 1). Intravenous amiodarone was initiated due to the debut of atrial fibrillation, and heart rate was 200 beats per minute. A transthoracic echocardiography revealed a hyperdynamic heart with normal left ventricular ejection fraction and without valve pathology or pericardial exudate. 

The severity of the condition with large skin detachment resulted in transfer to a specialized burn intensive care unit within 24 hours of admittance. Upon arrival at the intensive care unit, the patient was still in septic shock complicated by multiorgan failure. The patient was treated according to the burn unit protocol including fluid resuscitation using the Parkland formula with alternation of Ringers lactate and isotonic glucose. Shock reversal therapy was stopped after two days of treatment. Detachment counting 30% TBSA was observed, and specialized wound care was initiated. In addition, broad-spectrum antibiotics were given for a period of 10 days on an empirical basis consisting of meropenem, fucidin, and metronidazole intravenously combined with ocular chloramphenicol. Cultures came out negative except for expectorant *Candida* species. For supportive circulatory therapy, norepinephrine and dobutamine were administered, and renal failure was treated with CRRT (CVVHDF 25 mL/kg/hr). DIC was treated with a single dose of antithrombin 1500 IE and epoprostenol infusion (12 microgr/hr) for six days due to poor peripheral circulation and pulmonary shunting.

Punch biopsies did not support the diagnosis TEN, probably due to the delayed time of sampling corresponding to 7 days after admittance. Clinical symptoms were entirely compatible with TEN.

Slow clinical recovery was observed by supportive treatment without surgical intervention, and the patient weaned from the ventilator day 10 after admission. CRRT was continued for 12 days in total. The antibiotic therapy was modified guided by cultures from the tip of the central vein catheter presenting Gram-positive cocci, adding vancomycin to fucidin, whereas the other formulations were discontinued. After 18 days from hospital admission in total, of which 14 days were spent in intensive care unit, the patient was transferred back to the admitting hospital in near-habitual condition. The patient was discharged home on hospital day 19, only requiring ocular and paraclinical followup.

## 3. Discussion

TEN is a life-threatening exfoliative skin disease requiring urgent active therapy. The incidence is estimated at 1–3 people/million/year in Europe and the USA [1, 2, 9]. Mortality rates remain high despite technical advances and improvement in critical care [3]. Persons over 60 years seem to be more likely to develop TEN [7]. 

Numerous medications have been implicated as causes of TEN, and the most frequently associated drugs include aromatic anticonvulsants, sulfonamide antibiotics, allopurinol, nonsteroidal anti-inflammatory drugs, and the antiretroviral drug nevirapine [10].

Debut of cutaneous manifestations typically occur 1–4 weeks after intake of triggering drug. In this case, a very rapid onset was observed. The diagnosis relies on the one hand on clinical symptoms and on the other hand on histological features as well as excluding HUS and DRESS. The Nikolsky sign is not specific for SJS/TEN. Since the mechanism is not IgE mediated, a desensitization of the triggering drug is not an option [6]. Differential diagnoses include staphylococcal scalded skin syndrome (SSSS), acute generalized exanthematous pustulosis (AGEP), drug-induced pemphigoid, and pemphigus.

Infection is the most common cause of death in TEN patients and is often due to infection by *Staphylococcus aureus* or *Pseudomonas* species [11]. More than 50% of patients surviving TEN are suffering from long term sequelae primarily located on eyes, skin, and airways [2]. Recovery is slow and may require 3 to 6 weeks and is often accompanied by scarring at mucosal sites [1].

The mortality of TEN is high, but early admittance to a hospital burn unit has shown improved survival due to specialized care including fluid therapy and wound management in intensive care units with temperature regulated rooms available [10, 11]. Historically, systemic corticosteroids have been the standard of care despite the lack of randomized blinded trails supporting their efficacy in the treatment of TEN [12]. There is no evidence of the use of prophylactic empiric antibiotic therapy, since no survival advantages have been established [10, 11]. Plasmapheresis has been used with the aim of clearing drug metabolites and cytokines, and preliminary results have shown potential survival benefits performing anywhere from one to eight exchanges [10]. Treatment with high-dose IVIg (intravenous immunoglobulin) has shown promising results in the treatment of TEN [5]. Cyclosporine, cyclophosphamide, and anti-TNF-alpha antibodies are still controversial, but these treatments might provide benefit by blocking immune activation; however, currently, there is not enough evidence to draw conclusions on their effects [2, 5, 10]. There is no golden standard concerning wound care, and most centres follow local trends in burn care.

Because the pathophysiologic pathways and genetic predispositions have not yet been completely characterized, there is also a possibility that different treatment modalities and combinations for SJS/TEN might be effective at different stages of the disease or in different patient groups. For the last decade, the treatment of TEN has shifted away from steroid use in preference for high-dose IVIg. Despite the increased knowledge regarding the immunological aspects, only prompt withdrawal of suspect medication and general management of the patient as a burn patient are the only evidence-based therapies [10, 11]. 

The estimated mortality rate for this patient was >90%. We suppose that the successful outcome was caused by early recognition, termination of the suspect drug, early plasmapheresis, and aggressive treatment of septic shock during the whole period including CRRT. In addition, the rapid transfer to an intensive care unit specialized in burn treatment provided a positive impact on outcome tackling TEN-associated problems as well as the aggressive treatment of microbiological complication during the course. The patient received sepsis shock reversal therapy with corticosteroids the first two days of hospital admittance and not primarily as a treatment of TEN. Due to the positive reaction on treatment, experimental therapy with IVIg and TNF-alpha was not considered. 

## Figures and Tables

**Table 1:** SCORTEN severity-of-illness score (Bastuji-Garin [8]). The score of the patient is marked bold.

SCORTEN parameter	Individual score	SCORTEN sum (sum of scores)	Predicted mortality rate (%)
Age > 40 years	Yes = **1**, no = 0	0	3.2
Presence of cancer	Yes = 1, no = **0**	2	12.1
Heart rate > 120 beats per minute	Yes = **1**, no = 0	3	35.8
TBSA involved > 10%	Yes = **1**, no = 0	4	58.3
Serum urea level > 10 mmol/L	Yes = **1**, no = 0	>**5**	**90**
Plasma glucose level > 14 mmol/L	Yes = 1, no = **0**		
Bicarbonate level < 20 mmol/L	Yes = **1**, no = 0		
